# ^18^F-Fluorothymidine-Pet Imaging of Glioblastoma Multiforme: Effects of Radiation Therapy on Radiotracer Uptake and Molecular Biomarker Patterns

**DOI:** 10.1155/2013/796029

**Published:** 2013-04-04

**Authors:** Sanjay Chandrasekaran, Andrew Hollander, Xiangsheng Xu, Joseph L. Benci, James J. Davis, Jay F. Dorsey, Gary Kao

**Affiliations:** ^1^University of Washington School of Medicine, A-300 Health Sciences Center, Box 356340, Seattle, WA 98195, USA; ^2^Department of Radiation Oncology, University of Pennsylvania, 8-087 TRC, 3400 Civic Center Boulevard, Building 421, Philadelphia, PA 19104, USA

## Abstract

*Introduction*. PET imaging is a useful clinical tool for studying tumor progression and treatment effects. Conventional ^18^F-FDG-PET imaging is of limited usefulness for imaging Glioblastoma Multiforme (GBM) due to high levels of glucose uptake by normal brain and the resultant signal-to-noise intensity. ^18^F-Fluorothymidine (FLT) in contrast has shown promise for imaging GBM, as thymidine is taken up preferentially by proliferating cells. These studies were undertaken to investigate the effectiveness of ^18^F-FLT-PET in a GBM mouse model, especially after radiation therapy (RT), and its correlation with useful biomarkers, including proliferation and DNA damage. *Methods*. Nude/athymic mice with human GBM orthografts were assessed by microPET imaging with ^18^F-FDG and ^18^F-FLT. Patterns of tumor PET imaging were then compared to immunohistochemistry and immunofluorescence for markers of proliferation (Ki-67), DNA damage and repair (*γ*H2AX), hypoxia (HIF-1*α*), and angiogenesis (VEGF). *Results*. We confirmed that ^18^F-FLT-PET uptake is limited in healthy mice but enhanced in the intracranial tumors. Our data further demonstrate that ^18^F-FLT-PET imaging usefully reflects the inhibition of tumor by RT and correlates with changes in biomarker expression. *Conclusions*. ^18^F-FLT-PET imaging is a promising tumor imaging modality for GBM, including assessing RT effects and biologically relevant biomarkers.

## 1. Introduction 

Glioblastoma Multiforme (GBM) is a highly aggressive cancer that experiences a high rate of recurrence despite combination therapy [1]. There is significant need for tumor imaging modalities or biomarkers capable of identifying early stage tumors, posttreatment changes, and signs of tumor progression and recurrence of GBM [2]. Positron emission tomography (PET) is an imaging modality widely used in oncology for clinical staging, monitoring of treatment efficacy, and followup for disease recurrence [3, 4]. ^18^F-Fluorodeoxyglucose (^18^F-FDG) PET, which measures cellular glucose metabolism as a function of the enzyme hexokinase, is the most common clinically utilized PET modality. However, for imaging of high-grade brain tumors like GBM, ^18^F-FDG-PET imaging has limited utility. The high baseline level of glucose uptake within the brain results in a low signal-to-noise ratio and obscured tumor imaging [5, 6]. Consequently, several recent preclinical and clinical studies have investigated the use of alternative PET radiotracers, including ^18^F-Fluorothymidine (^18^F-FLT) and ^11^C-Methionine (^11^C-MET), for imaging of GBM [6–8].

^18^F-FLT, a nucleoside radiotracer, has emerged as a safe and promising tool to be used in conjunction with ^18^F-FDG-PET for studying aggressive and proliferative tumors like GBM [9, 10]. Thymidine uptake and metabolism require Thymidine Kinase-1 (TK-1) activity, which is elevated in the normal cellular S-phase and neoplastic tissues [11]. Additionally, unlike carbon-based thymidine, ^18^F-FLT has demonstrated higher specificity for TK-1 and reduced incorporation in DNA [12]. ^18^F-FLT-PET imaging has been shown in several clinical studies to correlate with cellular proliferation and tumor progression in high-grade glioma, as determined by the Ki-67 proliferation index [13–15]. Furthermore, Chen et al. demonstrated that increased radiotracer uptake in high-grade glioma was associated with reduced patient survival [13]. ^18^F-FLT-PET imaging has also been shown to correlate with treatment response in clinical studies of breast cancer and high-grade glioma [8, 16]. Similar studies have also been performed in other cancer types including lymphoma and soft tissue sarcoma [17–19]. We wished to study the effects of radiation on ^18^F-FLT-PET imaging of GBM tumors and secondarily effects on markers relevant to hypoxia, angiogenesis, and DNA damage and repair. Such data could provide additional critical information about treatment response and disease progression.

## 2. Materials and Methods

### 2.1. Radiotracers

^18^F-FLT and ^18^F-FDG were prepared at the University of Pennsylvania Small Animal Imaging Facility's (UPENN SAIF) PET Center cyclotron (Japan Steel Works; Model BC 3015; Tokyo, Japan).

### 2.2. Cell Culture

U251 GBM cell lines were engineered to stably express green fluorescent protein and luciferase (GFP-LUC) using pGreenFireTM Transcriptional Reporter Lentivectors purchased from System Biosciences (Mountain View, CA, USA) and sorted twice via flow assisted cell sorting (BD FACSVantage SE with DiVa software, San Jose, CA, USA at the University of Pennsylvania Flow Cytometry and Cell Sorting Center. Cells were grown in Dulbecco's Modified Eagle Medium (DMEM), supplemented with 10% fetal bovine serum and 1% streptomycin/penicillin antibiotics and maintained at 37°C in 5% CO_2_.

### 2.3. Mouse Tumor Model

All animal studies were conducted according to protocols approved by the University of Pennsylvania Institutional Animal Care and Use Committee (IAUCAC). Nude/athymic NCr (nu/nu) female mice were housed five per cage in a centralized small animal facility. Mice were checked continuously for good health, proper wound healing at the surgical site, and nondeformation of the cranium by the tumor.

#### 2.3.1. Heterotopic Flank Tumors

Six-week-old female nude/athymic mice were injected subcutaneously and bilaterally on the rear flanks with 2,000,000 U251-GFP-LUC GBM cells in 100 *μ*L of 1 : 1 media/Matrigel (BD Biosciences, San Jose, CA, USA). The expression of GFP and luciferase enabled two independent methods to distinguish the human tumor cells from the host mouse cells. First, mice were anesthetized by injection (IP) of ketamine (140 mg/kg) and xylazine (10 mg/kg). Upon receiving no response to a toe-pinch test, a 100 *μ*L suspension of cells, 1x phosphate buffered solution (PBS), and Matrigel were injected at a site superficial to the scapulae bilaterally and superficial and medial to the hip joints bilaterally [20]. After the procedure, the mice were returned to their cages and observed until the anesthesia had cleared and subsequently monitored on a daily basis for health, normal behavior, and visual tumor growth.

#### 2.3.2. Orthotopic Intracranial Tumors

Six-week-old nude/athymic mice were injected intracranially with 200,000 U251-GFP-LUC cells. First, mice were anesthetized by IP injection with ketamine (120 mg/kg) and xylazine (10 mg/kg). Additionally, mice received meloxicam (5 mg/kg) through IP injection in the preoperative and postoperative period.

Once confirmed to be unconscious by toe-pinch, each mouse individually was placed in a stereotactic head frame (Stoelting, Wood Dale, IL, USA). Once placed in the stereotactic frame, the operative area was decontaminated using betadine solution with a cotton tip applicator. All surgical procedures were performed using autoclaved or sterile instruments in a clean environment. First, a 1 cm midline scalp incision was made and the bregma was visualized. Next, a small burr hole was made through the skull using a Dremel drill 0.5 mm anterior and 2 mm lateral to the bregma as performed by Baumann et al. [21]. 200,000 cells in 10 *μ*L of 1xPBS were drawn into a Hamilton syringe. Then, the syringe was inserted into the arm of the stereotactic system with the 30G needle placed directly over the newly created burr hole. The syringe was inserted through the burr hole 3.3 mm from the skull surface. Cells were injected at a low rate (approximately 0.5 *μ*L/min) in order to minimize the pressure created at the injection site. Upon completion of the injection, the needle was removed, the burr hole was covered with bone wax, and the incision was closed using cyanoacrylate. After operation, the mice were returned to their cages and observed until the anesthesia had cleared and subsequently monitored on a daily basis for health, normal behavior, and tumor growth.

### 2.4. Mouse Tumor Imaging

#### 2.4.1. Bioluminescence Imaging (BLI)

The mice with tumors underwent weekly BLI to observe tumor growth and quantify tumor size [22]. BLI was conducted starting 1 week after tumor injection and continued on a weekly basis in a centrally shared IVIS Lumina II *In Vivo* Imaging System (Caliper Life Sciences; Hopkinton, MA, USA) according to the UPENN SAIF guidelines. First, the mice were anesthetized as described above (see Section 2.3.1) and confirmed by negative toe-pinch test. This was followed by dorsal, subcutaneous injection of 60 *μ*L of USP grade D-luciferin (D-Luciferin potassium salt; Gold Biotechnology; St. Louis, MO, USA). Imaging was conducted 5 min after D-luciferin injection over a period of 30 min to effectively determine the maximum luminescence intensity. Region of Interest (ROI) determination and analysis were conducted with the Living Image v4.0 software (Caliper Life Sciences; Hopkinton, MA, USA). Software-generated surface radiance measurements (photons/sec/cm^2^/steradian) were used to calculate Flux_max⁡_ values (photons/second) in order to determine tumor size. Tumors were allowed to grow without intervention until Flux_max⁡_ values approached 1 × 10^9^ (1*E* + 10).

#### 2.4.2. Radiation Therapy

Mice tumors with Flux_max⁡_ values on the order of 1 × 10^10^ photons/sec (~4–6 weeks after injection) were anesthetized via IP injection with ketamine (140 mg/kg) and xylazine (10 mg/kg). They then were individually irradiated with single-dose unilateral 16 Gray (Gy) radiation to the right side tumor in a department-owned unidirectional anterior-to-posterior beam X-Ray unit (Schneeman Electronics (Grants Pass, OR), model A-9002-100) with full body lead shielding except at the implanted tumor site and were then sacrificed 4–6 weeks later.

#### 2.4.3. MicroPET/CT Imaging

Experimental and control mice were subsequently followed by serial ^18^F-FLT-PET imaging. Both ^18^F-FDG and ^18^F-FLT microPET and microCT imaging were conducted at the UPENN SAIF laboratory. For flank tumors, ^18^F-FLT-PET microPET/CT was conducted prior to radiation therapy (RT) and 5 and 13 days (~1 week and 2 weeks) after RT. Mice were maintained on a 1–3% isoflurane and oxygen anesthesia system (VetEquip Inc; Pleasanton, CA) flowing at a rate of 1.0 liter/min. Radiotracer was delivered via a lateral tail vein injection with a maximum of 200 *μ*Ci–500 *μ*Ci and a 60 min uptake time. Animal temperature was maintained using a warm air source and subsequently monitored (Vetronics; West Lafayette, IN, USA). MicroPET imaging was performed on a Philips Medical System (Cleveland, OH, USA) small animal imaging machine (A-PET). Small animal microCT was subsequently performed on an ImTek scanner (CTI Molecular Imaging Inc. (now Siemens; Malvern, PA)) with a spatial resolution <50 mm. After imaging, mice were housed in a SAIF-designated area for handling radiotracers for 24 hours and monitored for radiotracer clearance before returning to the normal housing facility. MicroPET/CT images were retrieved in Analyze (.hdr) format. Radiotracer uptake was quantified by calculation of the percent of injected dose (%ID) of radiotracer located within an ellipsoid ROI. Total injected dose in mouse body, tumor, and brain were obtained via multiplication of the average ROI voxel value and size (mm^3^) using open-source Amide Medical Imaging Data Examiner (http://www.amide.sourceforge.net). CT bone and tissue reconstruction were conducted with open-source OsiriX Dicom Viewer (http://www.osirix-viewer.com).

### 2.5. Histological Analyses

#### 2.5.1. Tissue Harvesting

Mice were sacrificed with an overdose of ketamine and xylazine and perfused via a cardiac catheter with 1xPBS and 10% Formalin. Tumors were removed and stored at 4°C overnight in 10% Formalin and placed in a 30% sucrose solution, then frozen in cooled Isopentane (2-Methylbutane) and stored at −80°C.

#### 2.5.2. Immunohistochemistry

The tissue samples were sectioned into serial 10 *μ*m slices using a Microm cryostat (ThermoScientific; Walldorf, Germany) and placed on microscope slides (Thermo Fisher Scientific; Waltham, MA, USA). Slides were left at room temperature overnight and warmed for 30 min at 37°C prior to antigen retrieval. Slides were immersed for 15 minutes in citrate buffer titrated to pH 6.0 at 95°C, rinsed with 1xPBS, and membrane permeabilization was performed using Triton-X 100 (Sigma-Aldrich; St. Louis, MO, USA). Samples were stained for Ki-67 (BD Biosciences; San Jose, CA, USA), VEGF (Abcam; Cambridge, MA, USA), and HIF-1*α* (Santa Cruz; Santa Cruz, CA, USA) using an AB enzyme ABC kit (Vector Laboratories; Burlingame, CA, USA). Slides were incubated with the primary antibody for 60 min at room temperature, rinsed, and incubated with secondary antibody for 60 min at room temperature. AB enzyme was added with subsequent DAB Peroxidase Substrate (Vector Laboratories; Burlingame, CA, USA). Samples were fixed via immersion in an ethanol dilution series, final immersion in xylene, and sealed with Cytoseal (Thermo Scientific/Richard-Allan Scientific; Waltham, MA, USA).

#### 2.5.3. Immunofluorescence

Tissue samples were prepared as described above (see Section 2.5.2). Samples were stained for Ki-67 (BD Biosciences), VEGF (Abcam), HIF-1*α* (Santa Cruz), and *γ*H2AX (Millipore; Telecuma, CA, USA) using an ABC kit (Vector Laboratories, Burlingame, CA, USA) Slides were incubated with the primary antibody and then incubated with an AlexaFluor 594 conjugated (Invitrogen/LifeTechnologies; Carlsbad, CA, USA). secondary antibody. Slides were mounted using Mounting Medium with DAPI (Vector Laboratories). Samples were analyzed using a fluorescence microscope (Nikon Eclipse TE-2000U).

## 3. Results and Discussion

### 3.1. Intracranial MicroPET/CT Imaging

**Figure 1:**
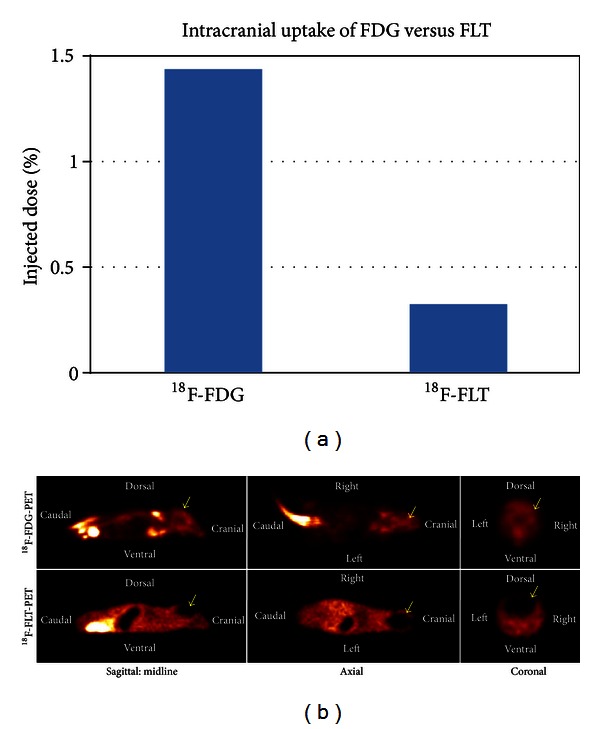
The normal brain shows intense uptake of FDG but not FLT. (a) Comparison of intracranial % injected dose (%ID) of ^18^F-Fluorodeoxyglucose (^18^F-FDG) and ^18^F-Fluorothymidine (^18^F-FLT) radiotracers in healthy nude/athymic mice. ^18^F-FDG uptake is greater than ^18^F-FLT in intact mouse brain. (b) Visual comparison of ^18^F-FDG-PET and ^18^F-FLT-PET imaging in nude/athymic mice for comparison of intracranial radiotracer uptake (yellow arrows).

To compare the patterns of uptake of ^18^F-FDG or ^18^F-FLT in healthy nude/athymic mice, the mice underwent microPET imaging 60 min following tail vein injection with either radiotracer. Intracranial radiotracer uptake was quantified based on % injected dose (%ID). ^18^F-FDG uptake into the normal brain was >4x higher than ^18^F-FLT uptake (%ID 1.43 versus 0.33, respectively) (Figure 1(a)). ^18^F-FDG uptake was uniform throughout the intracranial space when viewed in the coronal, axial, and sagittal planes, whereas ^18^F-FLT uptake was visually undetectable (Figure 1(b)). These results indicate that there is a high background signal with ^18^F-FDG that is not present for ^18^F-FLT. Concentration of both radiotracers within the bladder was noted to be comparable (data not shown). Such imaging validates that ^18^F-FLT uptake within the healthy brain is minimal compared to conventional ^18^F-FDG and suggests that intracranial ^18^F-FLT uptake would increase with tumor presence.

**Figure 2:**
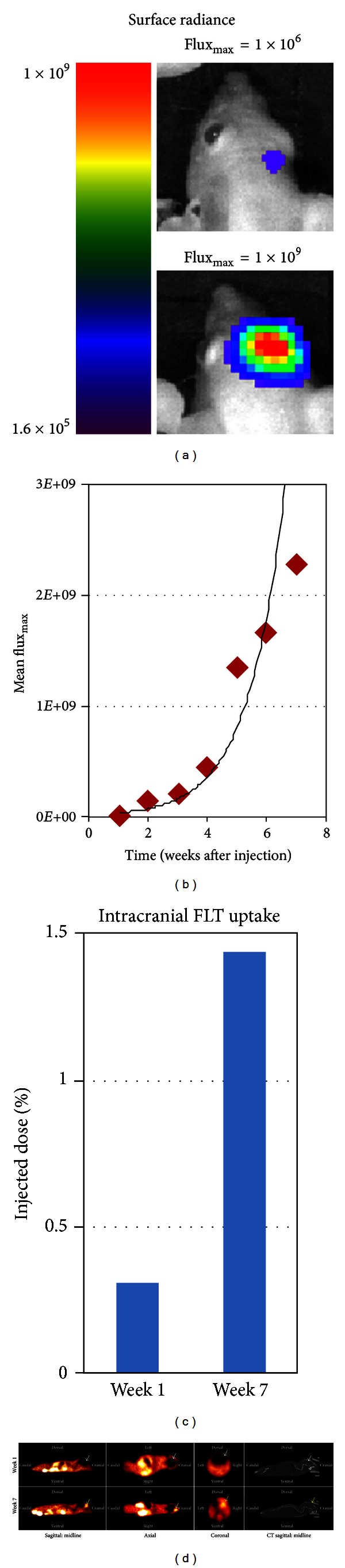
Orthotopic brain tumors are readily imaged by FLT-PET. (a) Visual comparison of Bioluminescence Imaging (BLI) surface radiance (photons/sec/cm^2^/steradian) in orthotopic intracranial tumors with BLI Flux_max⁡_ (photons/second) values of 1 × 10^6^ (1*E* + 6) and 1 × 10^9^ (1*E* + 9). (b) Identification of an exponential growth pattern in intracranial tumors based on Flux_max⁡_ measurements (*n* = 5). (c) Comparison of %ID of intracranial ^18^F-FLT %ID at 1 and 7 weeks after injection. (d) Visual comparison of ^18^F-FLT uptake in intracranial tumors based on Flux_max⁡_ values of 1 × 10^6^ and 1 × 10^9^ (weeks 1 and 7, respectively). Tumor visualized at 7 weeks (yellow arrows).

We thus compared uptake patterns of ^18^F-FDG and ^18^F-FLT in intracranial tumors. Healthy nude/athymic mice (*n* = 5) were intracranially injected with U251-GFP-LUC glioma cells and underwent weekly BLI with subsequent ^18^F-FLT-PET imaging (Figure 2(a)). Intracranial tumor growth across all mice demonstrated an exponential growth pattern as measured by changes in mean BLI Flux_max,_ with increased proliferation beyond 4 weeks (Figure 2(b)). ^18^F-FLT-PET imaging was performed in select mice (*n* = 2), and intracranial ^18^F-FLT uptake was quantified by %ID at weeks 1 and 7, which correlated with a BLI Flux_max,_ of 1 × 10^6^ and 1 × 10^9^, respectively. Radiotracer uptake was ~4.7x greater at 7 weeks versus 1 week (%ID 1.58 versus 0.33, resp.) (Figure 2(c)). Visually, radiotracer uptake was most appreciated in the larger intracranial tumors (7 weeks after injection). Additionally, radiotracer uptake at 7 weeks was localized to the tumor site, with minimal uptake in the surrounding normal brain tissue (Figure 2(d)). These data demonstrate that ^18^F-FLT concentrates preferentially within tumor and suggest that radiotracer uptake correlates with tumor size.

### 3.2. Flank Tumor MicroPET/CT Imaging

We wished to investigate the effect of radiation therapy (RT) on radiotracer uptake. However, the limited size of intracranial tumors in the mice did not permit sufficient imaging resolution. Additionally, mice with intracranial tumors with Flux_max⁡_ values greater than 1 × 10^9^ were quickly terminal (data not shown) and therefore unlikely to survive sufficiently long after irradiation for extended followup. Therefore, we resorted to flank xenografts for subsequent studies.

**Figure 3:**
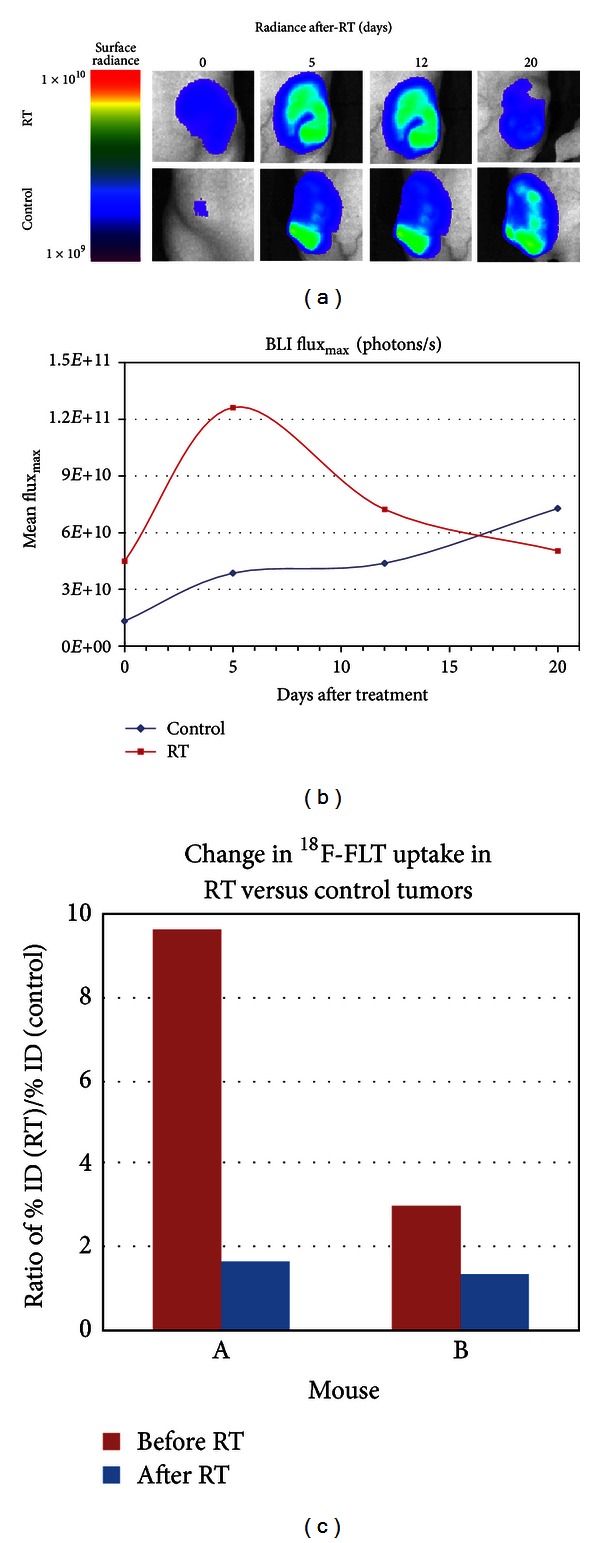
Radiation therapy leads to profound growth inhibition of flank tumors. (a) BLI of bilateral flank xenografts at 0, 5, 12, and 20 days following unilateral 16 gray (Gy) radiation therapy (RT). Reduced surface radiance noted in RT tumors. (b) Comparison of changes in BLI Flux_max⁡_ values in control (*n* = 3) and RT (*n* = 3) tumors. Reductions in Flux_max⁡_ seen by 5 days after RT. (c) Comparison of change in %ID of ^18^F-FLT uptake in RT versus control tumors (*n* = 2) before RT and 2 weeks after RT. RT tumors demonstrate decreased uptake at 2 weeks.

Experimental mice (*n* = 3) with bilateral U251-GFP-LUC flank tumors were exposed to a single unilateral fraction of 16 Gy RT to the right side flank tumor and tumor growth of the irradiated and control nonirradiated tumors was subsequently followed serially with BLI (Figure 3(a)). Flux_max⁡_ in control tumors progressively increased in the weeks following radiation, while RT tumors demonstrated reduction in BLI starting after 1 week (Figure 3(b)).

In addition to BLI, ^18^F-FLT-PET imaging of select experimental mice (*n* = 2) was performed before RT and 1 and 2 weeks following single dose 16 Gy RT of the right side flank tumor. Decreases in ^18^F-FLT radiotracer uptake in RT tumors were noted by 1 week after RT and sustained until 2 weeks after RT. Decreases in %ID in RT tumors at 2 weeks ranged from 23% to 64%. (Supplemental Figure 1, see Supplementary Material available online at http://dx.doi.org/10.1155/2013/796029) When comparing changes in %ID in RT versus control tumors, RT tumors exhibited significant reduction in radiotracer uptake by 2 weeks (Figure 3(c)).

**Figure 4:**
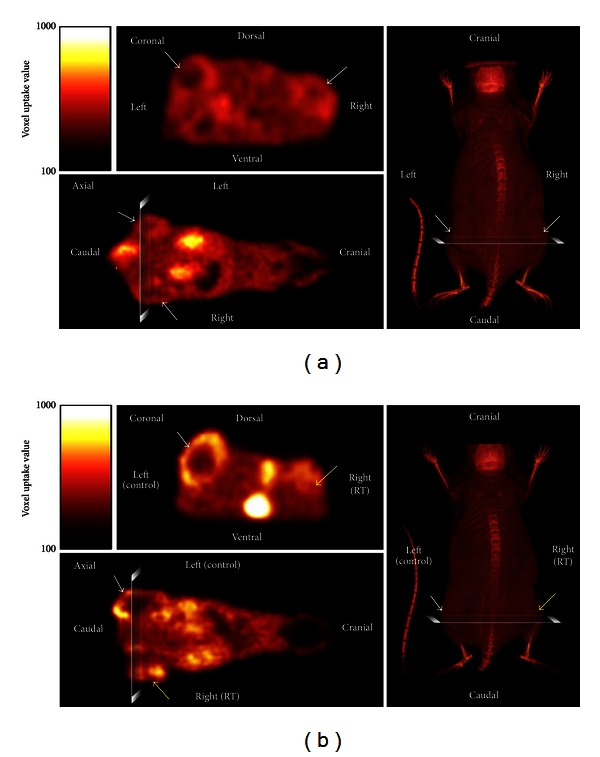
FLT-PET imaging demonstrates RT-induced changes in tumor cores. (a) ^18^F-FLT-PET of bilateral flank tumors prior to unilateral 16 Gy RT. Note the similar pattern of radiotracer uptake bilaterally in coronal and axial views (white arrows). 3D CT/bone imaging for flank surface anatomy is shown. (b) ^18^F-FLT-PET of bilateral flank tumors 2 weeks after unilateral 16 Gy RT. Unique variations in radiotracer uptake are seen. Note decreased radiotracer in control tumor core (white arrows); uniform uptake in RT tumors (yellow arrows). 3D CT/bone imaging for flank surface anatomy is shown.

Visually, ^18^F-FLT-PET imaging on both coronal and axial imaging suggests equal radiotracer enhancement between control and treatment tumors prior to RT (Figure 4(a)). Uptake patterns following radiotherapy, however, were different between treated and untreated tumors (Figure 4(b)). Left side control tumors were found to exhibit peripheral enhancement with notable central pallor, suggesting hypoxia induced central necrosis with the formation of pseudopalisades, a well-characterized phenomenon often seen in GBM [23, 24]. Quite strikingly, irradiated tumors were diminished in size, and the smaller tumors showed uniform radiotracer uptake throughout the tumor mass but with decreased intensity in comparison to control tumors.

For these pilot studies, a single dose RT regimen was utilized to investigate the effects of RT on tumor growth. Such a technique is similar to hypofractionated stereotactic radiosurgery which is used clinically for patients with cancer [25, 26]. These results in our mouse model encourage clinical follow-on studies with fractionated RT regimens.

### 3.3. Effects of Radiation Therapy on Cellular Proliferation and DNA Damage

**Figure 5:**
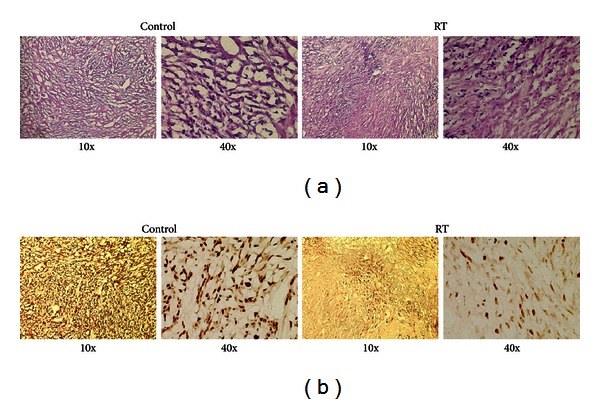
Radiation therapy leads to inhibition of proliferation. (a) Hematoxylin and Eosin (H&E) staining of control flank tumors and flank tumors receiving 16 Gy RT (10x and 40x). (b) Immunohistochemical staining for proliferation marker Ki-67 for flank tumors as performed in (a).

The decreased ^18^F-FLT-PET radiotracer uptake in RT tumors compared to control tumors seemed likely due to reduced tumor cell proliferation. To test this hypothesis, we performed immunohistochemistry (IHC) for Ki-67, an established marker for cellular proliferation. Mice with irradiated and control xenograft tumors were sacrificed, and the tumors were subsequently harvested and processed for histologic examination. Hematoxylin and Eosin (H&E) staining appeared comparable between control and irradiated tumors (Figure 5(a)). In contrast, irradiated tumors showed markedly decreased Ki-67 staining (Figure 5(b)). These results suggest that RT led to decreased tumor proliferation, thus reflected by diminished Ki-67 activity. Similar findings of diminished Ki-67 after radiation have also been noted in the clinic in both astrocytoma and oral squamous cell carcinoma [27, 28].

**Figure 6:**
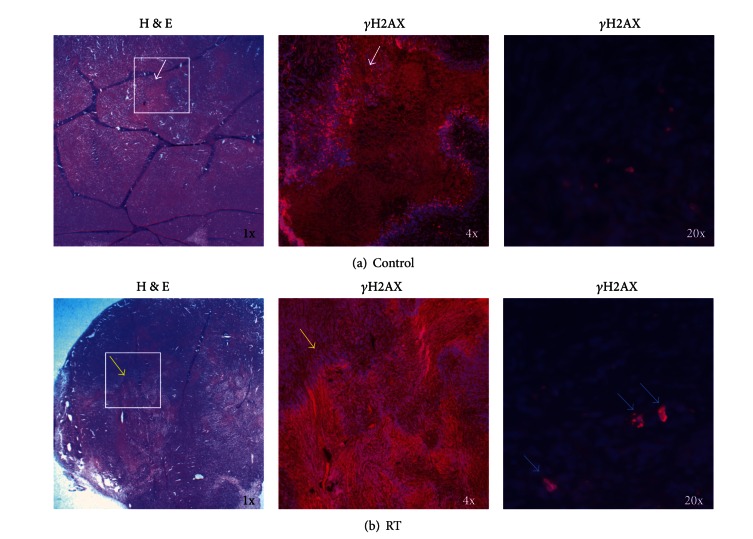
Radiation therapy leads to marked increase in DNA damage within the targeted tumor. (a) H&E stain (1x) and immunofluorescent staining w/ DAPI (blue) for Gamma-H2AX (*γ*H2AX) (red) (4x and 20x) in control flank tumors, with specific focus on tumor core (white arrows). (b) Staining as in (a) in flank tumors after 16 Gy RT, with specific focus on the tumor core (yellow arrows) and notable *γ*H2AX expression at 20x (blue arrows).

We also sought to evaluate if decreased tumor proliferation secondary to irradiation also corroborated with initiation of cell death. H&E staining was compared to Gamma-H2AX (*γ*H2AX) expression using immunofluorescence (IF). *γ*H2AX is a DNA histone that undergoes phosphorylation on the Ser139 residue in the presence of double-stranded DNA (dsDNA) breaks, such as that induced by RT. Increased *γ*H2AX signaling is suggestive of successful RT delivery [29–31]. Greater attention was given to expression levels within the tumor core, given that GBM is known to express phenotypic and genotypic heterogeneity in tumor markers at or within the tumor core [32, 33]. Control tumors demonstrated increased eosin staining within the tumor core (Figure 6(a)). These areas also correspond with reduced *γ*H2AX foci (red) and DAPI signaling (blue). Peripheral to the tumor core, multiple *γ*H2AX foci with corresponding DAPI staining are noted. Such findings suggest a lack of viable cells within the tumor core, likely due to tumor necrosis. Similarly, such necrotic foci have been demonstrated to be a hallmark of GBM on both pathology and radiology [34].

In irradiated tumors, H&E staining also revealed large, centrally located areas that are composed of a milieu of mixed hematoxylin and eosin localization (Figure 6(b)). In comparison to control tumors, IF studies for *γ*H2AX (red) expression in irradiated tumors demonstrated increased biomarker expression with positive DAPI (blue) staining of nuclei in within the central milieu. This is best visualized at higher resolution (20x). Positive *γ*H2AX expression and positive DAPI staining in these central areas are suggestive of post-irradiation cell recovery and repair in the setting of radiation induced apoptosis [35–37].

### 3.4. Impact of Radiation Therapy on ^18^F-FLT-PET Imaging, and Markers of Angiogenesis, and Hypoxia

The reduced proliferation of tumor and tumor necrosis may be due at least in part to RT effects on the tumor vasculature. There is evidence that GBM undergoes vascular changes relating to hypoxia and angiogenesis, and these often impact tumor growth and treatment resistance. Such findings have been visualized clinically with PET imaging [38]. We therefore sought to compare ^18^F-FLT-PET imaging to IF staining for markers for angiogenesis and hypoxia. Hypoxia is known to be involved in tumor recurrence, chemo- and radio-resistance, invasion, and decreased patient survival [24, 39]. We chose to examine expression of vascular endothelial growth factor (VEGF) and hypoxia induction factor-1*α* (HIF-1*α*), as both have been studied extensively and demonstrated to be active in GBM [24, 40–43].

**Figure 7:**
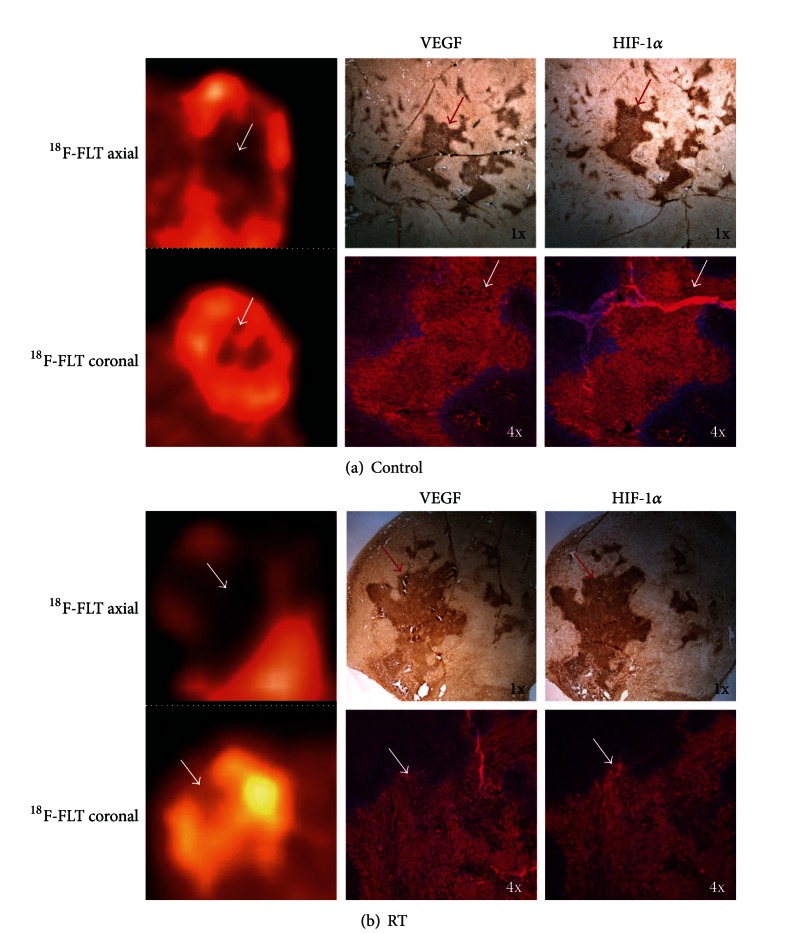
Decreased ^18^F-FLT uptake corresponds with regions of hypoxia and angiogenesis. (a) Comparison of axial and coronal views ^18^F-FLT-PET imaging sections in control flank tumors and immunohistochemical (IHC) and immunofluorescent (IF) staining with DAPI for vascular endothelial growth factor (VEGF) and hypoxia induction factor 1 alpha (HIF-1*α*). Positive VEGF and HIF-1*α* (red) with reduced DAPI (blue) is noted (white and red arrows). (b) Comparison of ^18^F-FLT-PET imaging sections and corresponding IHC and IF staining in flank tumors 2 weeks after 16 Gy RT as performed in (a). Positive tumor VEGF and HIF-1*α* expression with DAPI signaling are noted (white and red arrows).

^18^F-FLT-PET imaging of control tumors 2 weeks after unilateral RT demonstrated a noticeable reduction in radiotracer uptake within the tumor core as shown in both axial and coronal planes, potentially due to vascular insult (Figure 7(a)). IHC staining patterns for VEGF and HIF-1*α* (red) correlate with regions of increased eosin staining, as previously shown. Expression of both biomarkers may be mutually related, as increased hypoxia secondary to proliferation can induce VEGF production as a means to stimulate angiogenesis and reduce hypoxia [23, 34, 44]. IF staining reveals similarly absent DAPI (blue) staining with increased fluorescence for VEGF and HIF-1*α*. This superimposed central presence of VEGF and HIF-1*α* in nonirradiated tumor cores supports our prior hypothesis of central necrosis [24].

In irradiated tumors, ^18^F-FLT radiotracer uptake was also diminished within the tumor core, similar to control tumors (Figure 7(b)). IHC staining for VEGF and HIF-1*α* (red) was visually identical. IF staining of tumor cores was positive for increased expression of both biomarkers with concomitant positive DAPI (blue) signaling. Similar increases in VEGF expression after RT in GBM have been demonstrated *in vitro* [45]. Similarly, RT is known to induce hypoxia and to increase HIF-1*α* expression [46, 47]. Additionally, in C6 rat gliomas, HIF-1*α* upregulation was noted within 48 hours and up to 8 days following 8 Gy RT [48]. These data suggest that increased biomarker expression following high dose 16 Gy RT is secondary to ongoing tumor repair.

## 4. Conclusions

^18^F-FLT-PET imaging provides an exciting alternative to ^18^F-FDG-PET in the study of GBM. In our intracranial mouse model, we demonstrated successful ^18^F-FLT imaging of intracranial GBM orthotopic tumors with radiotracer sparing of the intact mouse brain. Additionally, ^18^F-FLT uptake within tumors correlated with tumor size.

In heterotopic flank xenografts, ^18^F-FLT radiotracer uptake patterns suggest irradiated tumors demonstrate detectable but globally diminished radiotracer uptake as seen by decrease in %ID. This is most likely due to tumor cell death and reduced postirradiation proliferation (Ki-67). Irradiated tumors also showed changes for biomarkers of dsDNA breaks (*γ*H2AX), hypoxia (HIF-1*α*), and vascular remodeling (VEGF).

These data suggest that ^18^F-FLT radiotracer uptake varies due to changes in the GBM tumor microenvironment, and that PET imaging may readily identify these changes. Such data encourage further *in vivo* and clinical studies with novel PET radiotracers such as ^18^F-FLT, with hopes of ultimately improving the imaging and care of patients with GBM.

## Supplementary Material

Figure 1: Changes in ^18^F-FLT flank tumor uptake seen at 1 and 2 weeks post-RT.
