# Supplementary material for: 18F-Fluorothymidine-Pet Imaging of Glioblastoma Multiforme: Effects of Radiation Therapy on Radiotracer Uptake and Molecular Biomarker Patterns
Source: ScientificWorldJournal. 2013 Apr 4;2013:796029. doi: 10.1155/2013/796029 (PMC3649687; doi:10.1155/2013/796029)
Supplement: Supplementary file 1 — Figure 1: Changes in 18F-FLT flank tumor uptake seen at 1 and 2 weeks post-RT. [file 796029.f1.pdf]

A.

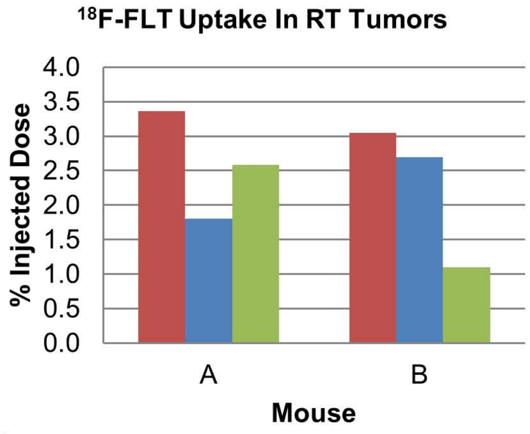

B.

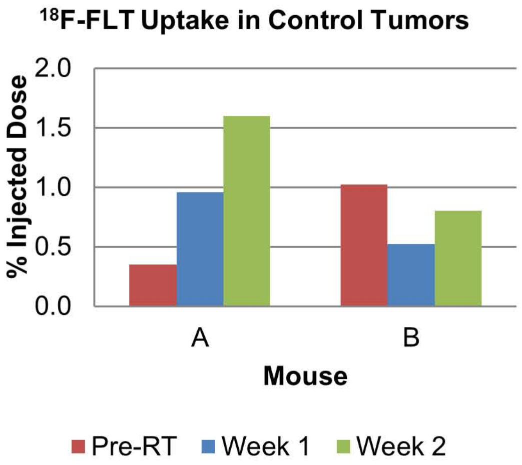

Supplemental Figure 1. Changes in ^{18}F-FLT flank tumor uptake seen at 1 and 2 weeks post-RT. (**A**) % ID of ^{18}F-FLT uptake in RT tumors (n=2) pre-RT and 1 & 2 weeks post-RT. Decrease in ^{18}F-FLT uptake seen by 1 week and persists until 2 weeks. (**B**) % ID of ^{18}F-FLT uptake in control tumors (n=2) pre-RT and 1 & 2 weeks post-RT. Tumors show increase or minor decrease in % ID.
